# Does Clubfoot Affect Sports Ability and Participation in Children?

**DOI:** 10.2106/JBJS.OA.26.00209

**Published:** 2026-08-18

**Authors:** Michelle Mo, Megan Hannon, Patricia E. Miller, Winnie Lin, Matthew Rauseo, Shawn Cameron, Akossiwa Assignon, Maria F. Canizares, William Meehan, Susan T. Mahan

**Affiliations:** 1 Department of Orthopaedic Surgery, Boston Children’s Hospital, Boston, Massachusetts; 2 Department of Orthopaedics, Harvard Medical School, Boston, Massachusetts

## Abstract

**Background:**

Patients and families of school children with unilateral corrected talipes equinovarus (TEV, idiopathic clubfoot) have anecdotally noted a difference in sporting function from the unaffected side to the clubfoot. The purpose of this study was to assess difference in strength, balance, performance, and patient perception of their sporting ability using contemporary sports research technology.

**Methods:**

Following Institutional Review Board approval, 27 unilateral Ponseti-treated patients with clubfoot (ages 8-15) enrolled in a cross-sectional study. Demographics and patient-reported outcomes (Patient Reported Outcomes Measurement Information System [PROMIS] and Oxford Ankle Foot Questionnaire for Children [OxAFQ-C]) were collected. Strength, range of motion, and functional mechanics were measured using dynamometry, markerless motion capture, and forceplates, respectively (VALD ForceFrame, HumanTrak, and ForceDecks). Comparisons between affected and unaffected limbs were conducted using paired Student’s *t* tests.

**Results:**

Mean evaluation age was 11.2 years (30% male); mean initial Dimeglio score was 10.5. Most (78%) participated in >2 sports. PROMIS Physical Activity mean score was 52.3 (SD 6.6), and mean OxAFQ-C Physical score was 81.5 (SD 17.5). Comparing TEV and unaffected limbs in range of motion and strength found differences in ankle dorsiflexion force (mean 58.4N [SD 20.8] vs. 68.6N [28.1]; p = 0.02) and lateral spine tilt during lunge stance (−0.2° [2.6] vs. −1.5° [2.4]); p = 0.04). No significant differences were noted with most performance measures, with small differences noted in single leg stand on the clubfoot limb (1005 mm vs. 781 mm), and mean velocity (67 mm/s vs. 52 mm/s; p < 0.001), jump height (1.6 in vs. 2.2 in; p < 0.001), and peak power (749W vs. 808W; p = 0.02).

**Conclusions:**

Children with unilateral TEV report favorably on standardized reports of patient-reported and functional assessments including sports participation. Although differences were noted with decreased balance with flexibility, single leg stance, and jump height on the clubfoot limb, overall, the magnitude of these differences was generally small, and their clinical significance remains uncertain given the lack of established minimal clinically important differences (MCIDs) for this population.

**Level of Evidence:**

Prognostic Level IV (cross-sectional, small sample size). See Instructions for Authors for a complete description of levels of evidence.

## Introduction

The accomplishments of Mia Hamm, Kristi Yamaguchi, and Troy Aikman are often cited as evidence for successful outcomes of clubfoot treatment. However, these high-profile examples do not clarify how the average child with clubfoot fares in terms of sports participation and functional performance.

Talipes Equinovarus (TEV), also known as clubfoot, affects approximately 1 to 4 in 1,000 live infants^1^. Clubfoot can occur bilaterally or unilaterally and affects the appearance of the foot but, particularly if untreated, the mobility, physical functionality, and quality of life of the child^1^. Some of these issues may persist even in well-corrected clubfoot^2^, and their effects on sports function remains unclear. The Ponseti method has substantially improved outcomes for most children with idiopathic clubfoot^2^, allowing them to lead normal lives. However, many families are concerned about potential limitations in athletic participation.

In patients with unilateral clubfoot, previous studies have shown residual asymmetries in strength, balance, and performance even after Ponseti treatment. The affected limb is often shorter, with reduced calf muscle bulk compared to the contralateral side^3,4^. Despite this, gross motor development typically progresses appropriately for age^5-7^ and Karol et al. found no difference in parent perceived physical function^5^. However, reduced activity levels have been noted in some cohorts^8^. While some asymmetry is expected, the extent of strength, balance, and gait differences in the sporting child with unilateral corrected clubfoot and its effect on patient reported outcomes (PROs) remains poorly characterized. The aims of this study were to (1) assess PROs and foot function in relation to physical activity and sports participation; (2) quantify static and dynamic asymmetry in strength and balance; and (3) evaluate gait asymmetry during walking and running. We further sought to determine whether asymmetry in strength, range of motion, balance, or gait was associated with lower physical activity scores and poorer PROs.

## Materials and Methods

After institutional review board approval, a cross-sectional study was performed at Boston Children's Hospital between February and July 2023. Of 320 screened registry patients, 27 were enrolled based on inclusion and exclusion criteria. Written parent consent and child assent were obtained before participation. Inclusion criteria were (1) unilateral idiopathic clubfoot, (2) treatment with the Ponseti method, (3) majority of clubfoot care at our institution, (4) age 8 to 15 years, (5) no clubfoot treatment within the preceding 6 months, and (6) no lower extremity injury within 6 months. Exclusion criteria included neuromuscular or syndromic clubfoot, active treatment for recurrence, unresolved lower extremity injury, or any comorbidity limiting physical activity as determined by a primary care provider. Patients unable to read or speak English were excluded because validated non-English outcome measures and research personnel. Treatment-related data were extracted from the medical record.

A physician, athletic trainer, biomechanist, and research personnel were present at each study session. Participants completed height/weight measurements before rotating through 6 testing stations. All measurement systems were calibrated according to manufacturer recommendations before data collection. Testing was performed using standardized protocols by trained personnel. The ForceFrame, ForceDecks, and HumanTrak systems have demonstrated acceptable reliability and validity for strength, balance, and movement performance in athletic populations (Appendix A). Participants received compensation.

Patient-reported outcomes were assessed using PROMIS Physical Activity and the Oxford Ankle Foot Questionnaire for Children (OxAFQ-C). Higher scores on both validated scales represent greater physical activity and better function, respectively.

### Statistical Methods

Patient and condition characteristics were summarized descriptively. Clinical range of motion, body movement, and gait measurements were summarized separately for affected and unaffected limbs among participants with complete paired measurements. Within-participant comparisons were conducted using paired two-sample Student’s *t* tests, with p-values adjusted for multiple comparisons using the Benjamini-Hochberg false discovery rate (FDR) method.

For unilateral measurements, percent difference was calculated as 100x(affected-unaffected)/unaffected. Percent differences and 95% confidence intervals (CI) were reported graphically. A percent different was considered statistically different if the 95% CI excluded 0. Spearman correlation evaluated associations between percent differences and PROs. Correlations remaining significant after FDR adjustment are displayed in the heatmap.

Source of funding: Internal funds supported this study. No external funds were used.

## Results

Twenty-seven patients (30% male) with unilateral TEV were analyzed at an average age of 11.2 years (SD, 2.1; Table I). The mean Dimeglio score at diagnosis was 10.5 (SD, 3.5). 82% of patients underwent heel cord tenotomy, 15% anterior tibialis tendon transfer, and 11% underwent other procedures including (1) open Achilles z-lengthening, posterior capsular release of the tibiotalar joint, and proximal tibia epiphysiodesis, (2) vulpius and right plantar fascia release, and (3) revision tendoachilles lengthening. Final clinic follow-up leg length discrepancy (LLD) measurements were available for 18 of 27 participants (66.7%), as these data were obtained from routine clinical follow-up and were not available for all participants at the time of study enrollment. Among those with available measurements, the mean LLD was 0.36 ± 0.24 inches (range, 0-0.875 inches). LLD was reported descriptively and was not included in the paired statistical analyses. Lastly, majority of patients participated in soccer (59%) or basketball (56%), with 78% of the cohort playing more than one sport (Table II).

**TABLE I:** Cohort Summary (*N* = 27)

Characteristic	Freq (%)
Age (*yr; mean [SD]*)	11.2 (2.1)
Sex (male; *% male*)	8 (30%)
Height (*m; mean [SD]*)	1.5 (0.2)
Weight (*kg; mean [SD]*)	43.7 (15.5)
Race	
White	16 (59%)
Black, African American/Canadian, or African	2 (7%)
Other	4 (15%)
Prefers not to answer	5 (19%)
Dimeglio score at presentation (*mean [SD]*)	10.5 (3.6)
Laterality (*% right*)	17 (63%)
Dominant foot	
Left	1 (4%)
Right	20 (74%)
Unknown	6 (22%)
Treatment	
Tenotomy	22 (82%)
Anterior tibialis tendon transfer	4 (16%)
Other	3 (11%)
PROMIS physical activity (*mean [SD]*)	52.3 (6.6)
OxAFQ-C (*mean [SD]*)	
Physical	81.5 (17.5)
School	97.2 (12.2)
Emotional	97 (12.1)

OxAFQ-C = Oxford Ankle Foot Questionnaire for Children.

**TABLE II:** Sports Participation

Sport	Freq (%)
Number of sports	
1	6 (22%)
2	5 (19%)
3	7 (26%)
4	2 (7%)
5	3 (11%)
6	3 (11%)
11	1 (4%)
**Sport**	**Freq** (**%**)
Soccer	16 (59%)
Basketball	15 (56%)
Baseball	9 (33%)
Football	9 (33%)
Other	7 (26%)
Swimming and diving	6 (22%)
Weightlifting	5 (19%)
Gymnastics	3 (11%)
Badminton	3 (11%)
Golf	2 (7%)
Downhill skiing	2 (7%)
Snowboarding	2 (7%)
Dance	2 (7%)
Competitive running	1 (4%)
Ice Hockey	1 (4%)
Lacrosse	1 (4%)
Tennis	1 (4%)
Squash	1 (4%)
Sailing	1 (4%)
Volleyball	1 (4%)
I do not participate in sports	1 (4%)

### Clinical Measures

**Fig. 1:**
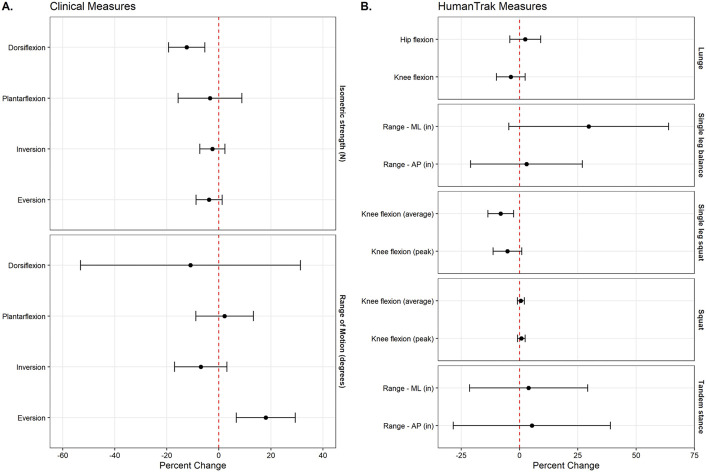
Mean percent difference between the TEV affected and unaffected limbs for (A) Goniometer and ForceFrame measures and (B) HumanTrak measures. Values were calculated as 100*(Affected-Unaffected)/Unaffected and presented with 95% confidence intervals. The dashed vertical line indicates no difference between limbs (0%). AP = anteroposterior, ML = mediolateral, and TEV = talipes equinovarus.

No significant differences were found between limbs with respect dorsiflexion, plantarflexion, or inversion to range of motion (all p > 0.05) (Table III). However, eversion range of motion was greater on the affected side (38° vs. 34°; percent difference, 18%; 95% CI, 7%-29%; p = 0.02) (Fig. 1A). Similarly, no differences were observed in isometric plantarflexion, inversion, or eversion (all p > 0.05). Isometric dorsiflexion strength was lower on the affected side (58.4 vs. 68.6; −12%; 95% CI, −19.0% to −5%; p = 0.02).

**TABLE III:** Comparisons in Measurements between Affected and Unaffected Sides

			Affected	Unaffected	
Tool	Test	Measure	Mean (SD)	Mean (SD)	P*
Goniometer	**Range of motion** (*degrees*)	Dorsiflexion	9.4 (8.6)	5.6 (13.8)	0.06
		Plantarflexion	45.0 (10.2)	43.5 (13.1)	0.60
		Inversion	38.2 (8.2)	43.3 (12.1)	0.06
		**Eversion**	**38.2** (**9.7**)	**33.5** (**9.5**)	**0.02**
Force frame	**Isometric strength** (*N*)	**Dorsiflexion**	**58.4** (**20.8**)	**68.6** (**28.1**)	**0.02**
		Plantarflexion	98.1 (45.6)	108.9 (60.1)	0.11
		Inversion	48.9 (19.7)	50.4 (19.8)	0.17
		Eversion	45.0 (17.7)	47.4 (19.1)	0.06
3D Motion analysis - humantrak	**Tandem stance** (*degrees*)	Range—ML (*in*)	0.76 (0.4)	1.96 (6.0)	0.46
		Range—AP (*in*)	1.02 (0.6)	1.38 (1.2)	0.24
		Pelvic lateral tilt	0.40 (1.6)	0.15 (1.6)	0.66
		Trunk lateral flexion	0.18 (1.5)	0.34 (1.4)	0.66
	**Lunge** (*degrees*)	Pelvic lateral tilt	2.1 (13.7)	3.3 (17.8)	0.46
		**Trunk lateral flexion**	**−0.17** (**2.0**)	**−1.51** (**2.4**)	**0.04**
		Hip flexion	76.19 (10.1)	75.99 (14.2)	0.94
		Knee flexion	95.29 (16.1)	99.77 (14.6)	0.25
	**Single leg balance** (*degrees*)	Range—ML (*in*)	0.93 (0.5)	0.81 (0.4)	0.40
		Range—AP (*in*)	1.21 (0.5)	1.52 (1.4)	0.40
		**Pelvic lateral tilt**	**7.4** (**2.5**)	**−7.9** (**3.8**)	**<0.001**
		**Trunk lateral flexion**	**4.7** (**2.9**)	**-4.8** (**3.0**)	**<0.001**
	**Single leg** **squat average measures** (*degrees*)	Trunk lateral flexion	1.8 (8.4)	-0.4 (5.5)	0.523
		**Knee flexion**	**72.7** (**14.2**)	**79.7** (**14.5**)	**<0.001**
		**Knee valgus**	**4.1** (**6.3**)	**12.5** (**10.8**)	**<0.001**
		**Knee varus**	**17.0** (**8.3**)	**8.2** (**7.3**)	**<0.001**
	**Single leg** **Squat peak measures** (*degrees*)	Trunk lateral flexion	4.6 (9.2)	1.8 (6.0)	0.46
		Knee flexion	79.2 (16.9)	84.4 (16.3)	0.11
		**Knee valgus**	**6.6** (**8.4**)	**18.3** (**15.3**)	**<0.001**
		**Knee varus**	**25.1** (**11.3**)	**12.3** (**10.4**)	**<0.001**
	**Squat** (**average**)	Knee flexion (*degrees*)	114.2 (19.6)	114.0 (21.6)	0.82
	**Squat** (**peak**)	Knee flexion (*degrees*)	119.4 (20.2)	118.9 (22.0)	0.66
Neuromuscular performance - forcedecks	**Single leg stand**	**Total excursion** (***mm***)	**1,005.0** (**216.0**)	**781.3** (**215.9**)	**<0.001**
		**Mean velocity** (***mm/s***)	**67.0** (**14.4**)	**52.1** (**14.4**)	**<0.001**
		CoP range ML (*mm*)	41.1 (24.1)	38.4 (22.7)	0.41
		CoP range AP (*mm*)	61.4 (25.0)	50.6 (14.7)	0.14
	**Single leg jump**	Concentric mean force (*N*)	565.7 (200.0)	569.1 (187.5)	0.76
		Eccentric mean force (*N*)	427.4 (152.3)	426.5 (152.3)	0.74
		**Jump height** (***in***)	**1.63** (**0.6**)	**2.20** (**0.8**)	**<0.001**
		**Peak power** (***W***)	**749.4** (**280.3**)	**808.4** (**280.1**)	**0.02**
	**Single leg hop**	Mean contact time (*ms*)	306.6 (96.1)	297.6 (80.8)	0.74
		Mean flight time (*ms*)	205.1 (75.7)	219.9 (108.3)	0.62
		Mean reactive strength index (*ms*)	0.74 (0.3)	0.79 (0.4)	0.70
	**Hop and return**	Peak first landing force (*N*)	1,051.8 (350.4)	1,106.0 (403.1)	0.05
		Peak takeoff force (*N*)	947.2 (338.5)	956.5 (336.8)	0.75
		Eccentric duration (*ms*)	278.5 (240.9)	199.4 (113.5)	0.14
	**Land and hold**	**Peak drop landing force** (***N***)	**1,417.9** (**696.5**)	**1,657.6** (**600.5**)	**0.03**
		Time to stabilization (*s*)	0.53 (0.2)	0.59 (0.2)	0.14
Gait analysis	**Walking gait**	Step length (*cm*)	42.6 (10.9)	41.5 (11.0)	0.08
		Step length/height (*% height*)	28.3 (6.1)	27.7 (6.3)	0.09
		Contact time (*ms*)	806.6 (105.8)	807.2 (103.2)	0.87
		Stance phase duration (*%*)	65.9 (2.0)	66.3 (2.2)	0.08
		Swing phase duration (*%*)	34.2 (2.0)	33.7 (2.2)	0.08
		Stride angle (*degrees*)	18.0 (7.7)	18.1 (7.6)	0.87
		Impact Gs (*G*)	1.6 (0.6)	1.7 (0.7)	0.54
	**Running gait**	Contact time (*ms*)	16.4 (5.9)	16.2 (4.9)	0.87
		Impact Gs (*G*)	9.2 (1.9)	9.9 (2.2)	0.08
		**Footstrike type**	**10.4** (**2.3**)	**9.4** (**2.2**)	**0.03**

AP = anterior-posterior, CoP = center of pressure, G = G-force, ML = medial-lateral, N = Newton, and W = watts.

* P-values are based on the Student *t* test and have been adjusted for multiple comparisons using the Benjamini and Hochberg method to control the false discovery rate. Bold entries indicates significance at the 5% level.

### 3D Motion Analysis—HumanTrak

In the tandem stance position, no differences were detected in mediolateral (ML) or anteroposterior (AP) range of motion, pelvic lateral tilt, or trunk lateral flexion (all p > 0.05) (Fig. 1B and Table III). Similarly, in the lunge position, no differences were observed for hip flexion, knee flexion, or pelvic lateral tilt (p > 0.05). A small difference was detected in lateral trunk flexion (−0.17° vs. −1.51°; p = 0.04). With values coded such that positive indicates lean toward the affected side, both conditions reflected a slight lean away from the affected side, with a greater lean away observed in the latter condition. During single leg balance, no differences were found in ML or AP range of motion (p > 0.05). However, lateral pelvic tilt (7.4° vs. −7.9°; p < 0.001) and lateral trunk flexion (4.7° vs. −4.8°; p < 0.001) differed significantly, indicating greater lean toward their affected side.

The largest asymmetries were observed during the single leg squat (Fig. 1B and Table III). Knee flexion was lower on the affected side (73° vs. 80°; p < 0.001). Knee valgus was reduced on the affected side for both peak (6.6° vs. 18.3°; p < 0.001) and average (4.1° vs. 12.5°; p < 0.001) measurements. Conversely, knee varus was greater on the affected side for peak (25.1° vs. 12.3°; p < 0.001) and average (17.0° vs. 8.2°; p < 0.001) measurements. No differences were observed during bilateral squat (p > 0.05).

#### Neuromuscular Performance—ForceDecks

**Fig. 2:**
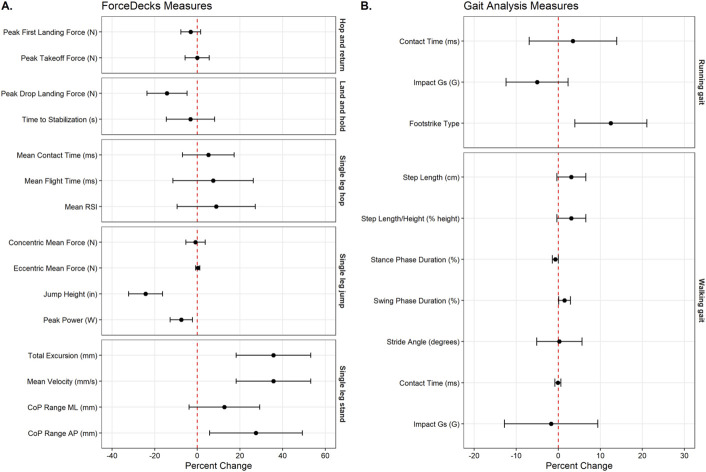
Mean percent difference between the TEV affected and unaffected limbs for (A) ForceDecks and (B) Gait analysis measures. Values were calculated as 100*(Affected-Unaffected)/Unaffected and presented with 95% confidence intervals. The dashed vertical line indicates no difference between limbs (0%). AP = anteroposterior, ML = mediolateral, and TEV = talipes equinovarus.

During single-leg stance, no significant differences were observed between limbs in ML or AP balance measures (p > 0.05). Although AP center of pressure (AP CoP) was greater on the affected side (61 mm vs. 51 mm), this did not reach statistical significance (p = 0.14). When expressed as percent difference, AP CoP was 28% higher on the effected side (95% CI, 6%-49%; p = 0.04) (Fig. 2A). Total excursion during single-leg stance was significantly greater on affected side (1005 mm vs. 781 mm; p < 0.001), indicating greater postural sway. Mean velocity was similarly higher (67 mm/s vs. 52 mm/s; p < 0.001), corresponding to a 36% increase (95% CI, 18%-53%; p < 0.001) for both measures (Fig. 2A). During the single-leg jump, no differences were detected in concentric or eccentric mean force (p > 0.05). However, jump height was lower on the affected side (1.6in vs. 2.2in; p < 0.001)., representing 24% reduction (95% CI, −32% to −17%; p < 0.001. Peak power was also modestly reduced (749W vs. 808W; −8%; 95% CI, −13% to −2%; p = 0.02) (Fig. 2A).

In the hop-and-return assessment, no differences were found in peak first landing force, peak takeoff force, or eccentric duration (p > 0.05). During the land-and-hold assessment, peak drop landing force was lower on the affected side (1418N vs. 1658N; −14%; 95% CI, −24% to −5%; p = 0.03). No additional differences were observed (Fig. 2A and Table II).

### Gait Analysis

During gait analysis, differences were observed between limbs in walking step length, contact time, stance duration, wing duration, stride angle, and impact force (all p > 0.05) (Fig. 2B and Table II). During running, no differences were found in contact time or impact force (p > 0.05). Footstrike type score was higher on the affected side (10.4 vs. 9.4; 13%; 95% CI, 4%-21%; p = 0.03), indicating a more anterior loading pattern at initial contact.

### Patient-Reported Outcomes

The mean PROMIS Physical Activity score for the cohort was 52.3 (SD 6.6). Mean OxAFQ-C domain scores included Physical 81.7 (SD 17.5), School 97.2 (SD 12.2), and Emotional 97 (SD 12.1). No significant associations were detected between any PRO domain score and measures of range of motion, isometric strength, or gait analysis.

## Discussion

This study evaluated preadolescent and adolescent athletes treated with the Ponseti method for unilateral clubfoot using a comprehensive sports-specific assessment comparing affected and unaffected limbs. We also used standardized PRO measurements to contextualize function relative to normative populations. Overall, most children reported high levels of sports participation and favorable PRO scores. Objective testing demonstrated predominantly symmetric performance across static strength, range of motion, and gait parameters with only subtle differences in dorsiflexion, jumping performance, and balance. These findings suggest that most youth treated for unilateral clubfoot demonstrated high-level functional symmetry during sports-specific tasks. This objective characterization of athletic performance provides clinically relevant data to guide counseling regarding sports participation, expectations, and injury prevention.

PROMIS Physical Activity scores were slightly above the population mean (52.3; SD 6.6)^9^, and OxAFQ-C scores were high across Physical 81.7 (SD 17.5), School 97.2 (SD 12.2), and Emotional 97 (SD 12.1) domains. Compared with previously reported baseline scores, our cohort demonstrated higher self-reported function across domains^10^. Our cohort participated in several sports, including running and jumping sports, with only 1 child reporting no sports participation. Previous cohorts have similarly reported limitations in activity and performance. Pavone et al.^11^ assessed 25 patients with 44 clubfeet treated by Ponseti method and found Clubfoot Assessment Protocol scores of 97.5 ± 6.4, American Orthopaedic Foot and Ankle Scores (Ankle-Hindfoot) of 97.5 ± 5.8, Foot and Ankle Disability Index (FADI) of 99.9 ± 0.6, and FADI Sport scores of 100. They reported no difference in male and female athletes, or between those with unilateral vs. bilateral clubfoot, and noted no limitations in sports participation or performance^11^. El-Banna et al.^12^ compared children treated for relapsed clubfoot with those without and found no difference in Clubfoot Disease Specific Instrument outcome. They also noted that children with clubfoot had step counts similar to children without clubfoot^12^. Mir et al.^13^ found that children with clubfoot were all participating in extracurricular sporting activities, with 80% of the children active 4 to 7 days a week compared with just 17% of the age-matched general population^13^.

Objective testing revealed selective differences consistent with the prior literature. In our cohort, dorsiflexion strength was reduced on the affected side, aligning with previous gait analyses demonstrating diminished ankle dorsiflexion following Ponseti treatment. Grin et al.^14^ reported significantly lower maximum dorsiflexion during swing phase compared with healthy controls (mean difference −2.17^◦^), as well as an increase in in-toeing during gait. Similarly, Karol and Jean^15^ described persistent abnormalities in ankle motion in children treated for clubfoot, including 43% with limited dorsiflexion and altered foot progression during gait. Some excessive dorsiflexion was noted in the younger population, comprising children younger than 5 years. They also noted that the clubfoot side was more likely to intoe^15^. Although prior studies assessed dynamic internal foot progression during gait rather than the static eversion observed in our cohort, this distinction may reflect persistent effects of Ponseti abduction bracing in older children.

Several measures suggest reduced balance on the clubfoot side, including sway in single-leg stance, altered trunk lateral flexion during lunging and squatting, and knee alignment (varus and valgus) during squat. Prior studies similarly report balance deviations in children with clubfoot. Lööf et al.^16^ observed increase stance and hopping deviations and suggested compensatory adaptation of the unaffected limb. A systematic review by Gosse et al.^7^ found lower balance domain scores for children with clubfoot, and Zapata et al.^17^ reported decreased balance despite otherwise normal gross motor development.

Measures of power were also reduced on the clubfoot side, including decreased single-leg jump height, peak power, and drop landing force. Prior gait analyses have reported diminished ankle power generation and reduced gastrocnemius strength in clubfoot limbs^14,15^. Lööf et al. found more than half the children with clubfoot had deviations in toe walking, heel walking, standing, and hopping on 1 foot^16^. Although many earlier cohorts were younger than ours, these findings suggest persistent, albeit subtle, deficits in balance and power.

Interestingly, despite measurable differences in flexibility, balance, and power, we found no significant correlations between limb asymmetry and PROs. Children with greater asymmetry did not report more difficulty with sports participation or have a reported difference in selection of sports activities. These findings suggest that subtle performance differences may not meaningfully impact perceived functional ability and provide objective data to guide counseling regarding expectations and injury prevention.

This study has several limitations. Although prior work has evaluated clubfoot using gait analysis, this is the first to use sport-specific range of motion assessment using markerless motion capture and isometric dynamometry in this population. We also note that although certain findings are significant, their immediate clinical relevance should be interpreted with caution as a validated minimum clinically important difference for the measurements described in this specific population has not yet been established in the literature. The relatively small sample size may also have limited detection of more subtle differences, and the small sample size also limits drawing meaningful conclusions regarding sport-specific participation patterns. In addition, the cohort included a higher proportion of female participants than typically seen in clubfoot populations, and the impact of that imbalance is not clear. Finally, participant self-selection may have introduced sampling bias.

Overall, youth treated for clubfoot demonstrated high rates of sports participation and favorable PROs. Although subtle differences in balance, flexibility, and jump performance were observed on the clubfoot side, performance was largely symmetric between limbs. Targeted physical therapy may help address these residual differences and further optimize function. Future studies examining sports-related injury risk in this population may clarify the impact of these findings.

## Appendix

Supporting material provided by the authors is posted with the online version of this article as a data supplement at jbjs.org (https://links.lww.com/JBJSOA/B305). This content was not copy-edited or verified by JBJS.
